# Understanding infection prevention behaviour in maternity wards: A mixed-methods analysis of hand hygiene in Zanzibar

**DOI:** 10.1016/j.socscimed.2020.113543

**Published:** 2021-03

**Authors:** Mícheál de Barra, Giorgia Gon, Susannah Woodd, Wendy J. Graham, Marijn de Bruin, Catherine Kahabuka, A. Jess Williams, Khadidja Konate, Said M. Ali, Rukaiya Said, Loveday Penn-Kekana

**Affiliations:** a University of Aberdeen, UK; b Brunel University London, UK; c London School of Hygiene and Tropical Medicine, UK; d IQ Healthcare, Radboud University Medical Centre, the Netherlands; e CSK Research Solutions, Tanzania; f University of Birmingham, UK; g Public Health Laboratory Ivo de Carneri, Zanzibar; h Ministry of Health Zanzibar, Zanzibar

**Keywords:** Risk of hospital-acquired infection, Hospital hygiene, Infection prevention and control, Health care professionals, Qualitative approach

## Abstract

**Rationale:**

Although women in low- and middle-income countries are increasingly encouraged to give birth at facilities, healthcare-associated infection of both the mother and newborn remain common. An important cause of infection is poor hand hygiene. There is a need to understand how environmental, behavioural, and organisational factors influence hygiene practice.

**Objective:**

To understand variations between facilities and between people in hygiene behaviour and to explore potential intervention targets in four labour wards in Zanzibar.

**Methods:**

Site visits including observation of deliveries and of day-to-day workings of the facilities. Thirty-three semi-structured interviews, totalling more than 46 hours, with birth attendants, orderlies, managerial staff and mothers. Transcribed interviews and observation notes were read and coded by two authors. Themes were developed and analysed in light of existing research.

**Results:**

The physical preconditions for hand hygiene were met more regularly in the two highvolume facilities, where soap, water, gloves were almost always available. However, in all of the facilities, hand hygiene appeared impeded by poor ergonomics, like, for example, physical distance between water taps, gloves, or delivery beds. Recontamination of gloved hands following good hand hygiene was commonly observed, a pattern that the birth attendants attributed to high and unpredictable workload and equipment shortages. Interviews and focus groups suggested that birth attendants typically understood when and why hand hygiene should be implemented, and that they were aware of low handwashing rates among co-workers. In poorer performing facilities, managers were less inclined to visit wards and more likely to perceive hand hygiene as beyond their influence.

**Conclusions:**

Observations and interviews suggest improvements in the ergonomic design of delivery rooms, including convenient availability of sinks, soap, hand gel, hand towels and gloves, may be a low-cost way to reduce the infection burden from poor hand hygiene.

## Highlights

• We ran a mixed-methods study on clean birth practices in Zanzibar.
• We interviewed 34 staff, observed 15 births, and spent 15 days in four facilities.
• While gloves, soap, and handgel are often present, they are rarely *convenient.*
• Midwives realise hygiene is low among colleagues not prioritised by management.
• Improved ergonomics and norm change may help improve infection control.

## 1 Introduction

About one in 13 neonates in lower- and middle-income countries acquire a severe bacterial infection (Seale et al., 2014) and an estimated one in 11 maternal deaths can be attributed to bacterial infection (Kassebaum et al., 2014). The global trend towards institutional delivery over home deliveries presents a significant opportunity to reduce morbidity and mortality associated with childbirth, including by enhancing infection prevention systems in these settings (Campbell et al., 2016).

Health care worker hand-hygiene during labour and delivery has long been recognised as an important infection reduction strategy (Ellingson et al., 2014; Gould, 2010). While hand hygiene rates before aseptic procedures during delivery and labour have rarely been measured in robust, replicable ways, evidence from a recent systematic review of hand hygiene before procedures during labour and delivery suggested rates of 1%–28% (Gon, 2019). Similarly, a systematic review of compliance to hand hygiene guidelines before patient contact in higher-income countries estimated compliance rates at 21% (Erasmus et al., 2010).

There is therefore a need for feasible and effective interventions that improve hand hygiene and reduce the burden of preventable bacterial infections in both newborns and mothers. One obvious and necessary approach is to improve water, sanitation, and hygiene infrastructure. Kruk et al. (2016) used Service Provision Assessments data to examine the health facilities in maternity wards in five African countries including Tanzania and found many primary and secondary care facilities lacked safe water and infection control resources. However, existing research indicates that while many facilities in Zanzibar – the site of the current study – have the infrastructure needed to implement hand hygiene, hand hygiene rates remain low (Gon et al., 2017). Our recent quantitative time-and-motion study, conducted as part of the HANDS (Hand-hygiene of Attendants for Newborn Deliveries and Survival) study, found that birth attendants performed inadequate hand hygiene before 90% of 781 observed procedures (Gon et al., 2018). Birth attendants did not perform one or more of the following steps adequately before the majority of procedures: apply sanitizer/wash with soap, avoid hand recontamination, don gloves, or avoid glove recontamination. Data collected as part of this project also indicate substantial differences between facilities in the rates of hand hygiene (Gon, 2019). These and previous findings suggest a need to understand the reasons why hand hygiene rates vary across facilities and to develop and implement interventions to improve hand hygiene in low and middle-income countries. This need is particularly pressing given ongoing encouragement of mothers to deliver in facilities rather than at home (Campbell et al., 2016).

The layout and organisation of delivery rooms may play an important role in facilitating or impeding hygiene and infection control. While the effects of water shortages or broken taps are obvious, there may be a more subtle relationship between infection control and the ergonomics (i.e., organisation and design) of the delivery room. For example, even small increases in the distance between patient and sink can decrease handwash rates (Deyneko et al., 2016). Moreover, there is a wealth of research suggesting that hospital design and layout can influence the safety and satisfaction of both staff and patients (Ulrich et al., 2008). Much of the maternity ward design literature has been conducted in high-income countries and focusses on the emotions and wellbeing of mothers and partners during the birthing process (Foureur et al., 2010; Hammond et al., 2014). However, while the effects of good ward design on wellbeing are important for mothers everywhere, the effects on infection control are particularly pertinent where maternal and newborn infections are common problems. A recent review of determinants of clean birthing practices (Esteves Mills et al., 2020) in low and middle-income countries found just two studies that refer to the layout and ease of use of hygiene materials in maternity wards, with one noting sinks are not well placed (Asp et al., 2011) and another reporting that midwives sometimes attribute a lack of handwashing to inconvenience (Ji et al., 2005). More detailed examination of the relationship between ward layout and infection prevention is thus warranted.

The current article arises from a sub-study of *HANDS,* a large multi-method project aimed at understanding hand hygiene in maternity wards. Site visits, that is, multi-day visits by a team of researchers to several sites (Yin, 2016), were selected over a longer-term embedded participant-observation since our approach enables comparisons across multiple settings (Chan et al., 1994), a goal of our project. The approach taken was qualitative and observational, and involved interviews, focus groups, and observation of hygiene behaviour. We also documented elements of the facilities’ physical and institutional design relevant to hygiene and infection control. These qualitative methods can offer unique insights into hygiene by observing behaviour in context and by allowing staff members to reflect upon and share their attitudes, beliefs, and observations about hand hygiene. Moreover, observational and qualitative research can play an important role in the intervention development process (Eldredge et al., 2016; Grol et al., 2013).

The present study has four objectives. Our first objective is to describe the infrastructure, organisation, and workload of the four facilities studied. This overview provides context for the subsequent analyses. Our second objective is to describe how differences between facilities in ergonomics, layout, and organisation appeared to enable or obstruct hygiene behaviour in the delivery rooms. Our third objective is to examine hygiene in the light of differences and similarities in how consumables like gloves, soap, and drying materials are used, both across individuals and facilities. In doing so, we draw attention to features of hygiene that have been underexplored in the literature but may have important implications for infection rates. These include the recontamination of gloves before procedures and the post-delivery cleaning of mothers with their own soiled cloths. Our final objective is to explore the social context of hygiene examining, for example, the normative status of handwashing and the influence of managers and other staff members. As advocated by George et al. (2018), we aim to go beyond seeing health workers as faceless numbers of units of health producers but instead recognising the importance of “their identities and motivations, daily routines and negotiations, and training and working environments”.

## 2 Methods

Setting. The study included two facilities on Unguja island and two facilities on Pemba island. Along with several much smaller islands, Unguja and Pemba form Zanzibar, a Muslim majority, semi-autonomous region with 3% of the Tanzanian population. Facilities A and B were larger, with several departments (surgery, paediatrics, etc.). Facilities C and D were smaller and focused primarily on maternity and outpatient services.

**Fig. 1:**
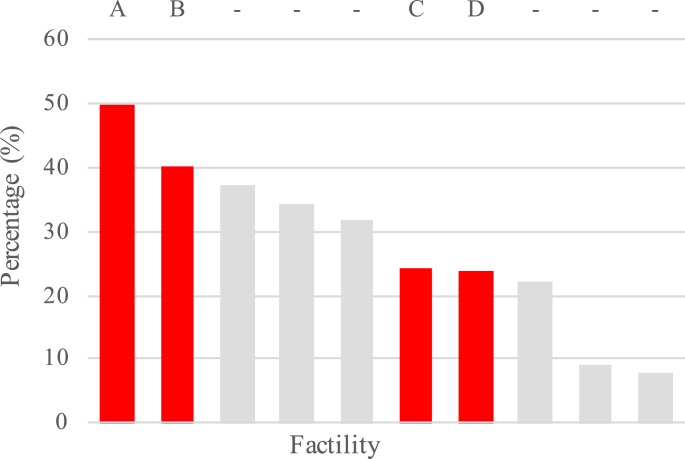
Average percentage compliance with hand hygiene before aseptic procedures in 10 facilities including the four facilities examined in the present study (A-D; adapted from Gon, 2019).

**Site and participant sampling.** The four facilities were selected from a sample of 10 which were included in the HANDS project. These four facilities were chosen to reflect the diversity across the delivery-volume spectrum, as well as an urban and rural spread. Quantitative assessment of hand hygiene in the 10 facilities suggests that these four facilities did vary in hand hygiene compliance (see Fig. 1).

Our visits were timed to coincide with shift ends, a convenient time for interviews, and birth attendant interviewees were selected based on their availability during these hospital visits. Since birth attendants’ cycle through shifts, this convenience sampling strategy approximates random sampling. Facilities typically had one maintenance person and ward manager, and thus no sampling took place at the within-facility level for these types of participants.

Participants. Birth attendants – here encompassing midwives and, in some facilities, orderlies/cleaners who deliver babies – were of primary interest, and three to four were interviewed per facility. The role of the midwives is to manage standard deliveries including antenatal and postnatal care, complete relevant paperwork, identify complications during pregnancy, perform appropriate interventions and where necessary, refer the mother or baby to other health care workers with the relevant expertise. The role of orderlies who deliver babies typically excludes paperwork and complicated deliveries. In some facilities, orderlies are not permitted to deliver while in others they frequently do so, though this role is not always explicitly acknowledged. Since the senior staff can influence hand hygiene both through organizing a consistent supply of hygiene consumables and also by creating the workplace norms, rules, and expectations, we also interviewed ward managers, hospital, and district level management. Finally, in the three facilities with a functional Infection Prevention Committee, we conducted a focus group discussion with the available members. The complete sample is described in Table 1.

**Table 1:** Data sources by facility and source.

		Number	Total Duration of interviews (min)
Interviews per facility			
	Facility A	10	432
	Facility B	10	270^a^
	Facility C	10	340
	Facility D	9	270
	District/regional level	3	100
Interviews per profession			
	Nurse birth attendants	11	372^a^
	Orderly birth attendants	2	83
	Infection control committees^b^	3	150
	Wash Maintenance Controllers	4	143
	Hospital managers	5	284
	District/Regional level supervisor	2	68
	Patron/matron	2	60
	Ward Manager	4	242
Observational data		Facility A,B,C,D	
	Structured observations	3,2,3,2	~300
	Deliveries observed	5,1,0,3	na
	Vaginal exams observed	5,2,2,2	na
	Days team spent in facilities	5,3,4,3	na

Note

a Excludes one untimed birth attendant interview.

b Focus group discussions rather than interviews.

**Site visits:** Our research team spent 3–5 days visiting each facility, during which we engaged in a range of qualitative practices, including interviews which are described in a separate subsection below. The visit team included two experienced, Swahili-speaking qualitative researchers, one of whom was medically trained, and, on some occasions, two behavioural scientists with backgrounds in infection control. First, we observed the day-to-day workings of the ward, established a rapport with staff members and considered what kind of more detailed observations might be conducted. Although external visitors and observers as well as foreign doctors are reasonably common in the hospital sites, some changes to staff behaviour as a consequence of our presence was likely.

During the visits, we noted labour ward activity by birth attendants, consumable use, and the organisation and use of space within these rooms. Maps were created of each facility and the location of all hygiene-related infrastructure and consumables were noted. We also noted how staff members interacted with each other and with the mothers (e.g., who assists who? What supervision exists? Are there formal or change-of-shift meetings? What happens during discharge?). We paid particular attention to the delivery procedures, newborn care immediately after birth, and the management of infection risks during this process. Other tasks included organizing interviews, observing between-delivery preparation and cleaning, generally becoming acquainted with the facility and its staff members, asking questions about the layout and organisation, and observing daily life in the facility. This ethnographic approach was complemented by semi-structured observations.

**Semi-structured observations:** Semi-structured observations were conducted in each facility in 30-min sessions. During these sessions, a researcher sat in the delivery room and observed one staff member. The researcher took detailed time-stamped notes on all hand-hygiene-related behaviour (handwashing, glove use, recontamination, etc.) and on the broader behaviour patterns of which the hygiene was a part (delivery, cord-cutting, vaginal exams, disposal of wastes, cleaning, delivery kit preparation, data entry, surface contact, colleague interaction, etc.). Semi-structured observation sessions were timed to coincide with deliveries or vaginal exams, and the focal staff member was chosen on the basis that they were ones who were delivering the baby or conducting the vaginal exam. These observations noted any deviations from the WHO-recommended hygiene practices. These are detailed elsewhere (World Health Organization, 2009, 2015), but the most relevant details are as follows: Hands should be cleaned directly before and after any contact with the woman or newborn, any time there is contact with blood or other body fluids, and after removing gloves. Hands should be cleaned by washing with soap and water if visibly contaminated; otherwise, both soap and water or with alcohol-based formulation are suitable. Gloves are to be worn in any procedure involving blood or body fluids including delivery and vaginal exam. The delivery should take place on a clean surface. To avoid recontamination of hands post handwashing, sterile and clean equipment must be prepared and laid out such that it can be accessed during the procedure.

**Interviews:** The interview topic guides themes were derived from the constructs in integrated behavioural theory (Eldredge et al., 2016), social norm theory (Bicchieri et al., 2014) and WHO hygiene guidelines (World Health Organization, 2009, 2015). Additional topics were added based on other hand hygiene studies as well as our observations of hygiene in the maternity wards. Interviews were conducted in Kiswahili. We asked about interviewee's own behaviour and about their perceptions of other staff members handwashing (e.g., how if 10 of your colleagues were to perform procedure X, how many do you think would wash hands afterwards?).

**Analysis:** The interviews were audio-recorded, transcribed, and translated into English. We adopted a generalised qualitative approach in which the transcripts were disassembled into low-level descriptive codes and then reassembled into themes that may help explain the observed behaviour (Yin, 2016). The development of the codes was a two-step process, assisted by NVivo 11 software. First, all transcripts and observation notes were read by a minimum of two authors, and the codes were deductively developed through discussion and reflection. We then compiled these codes and jointly applied them to a subset of five interviews. During this initial application of the codes, the definition, scope, and number of the codes evolved. Once the broader team confirmed their agreement on these new definitions, the 36 codes were then applied to the remainder of the transcripts, with some minor modifications occurring throughout the process. Example codes included “descriptions of glove use”, “influence of management”, “norms and sanctions”, “midwifes’ intervention ideas”. These codes were applied to the observation notes as well as to the interview transcripts so that reassembly and interpretation were based on both observations and transcripts.

Our selection of themes *–* defined here to include processes, spaces, or materials that may influence infection risk and that may be amenable to change – was informed by several factors. Firstly, it was informed by behaviour we observed during structured observations of deliveries and the ward more generally. Four authors visited one or more of the wards. These visits drew our attention to factors like ward ergonomics and the role of management. Theme selection was also informed by existing theory on behaviour change that might be relevant to hospital contexts (Bicchieri et al., 2014; Eldredge et al., 2016). Finally, and most importantly, the theme selection was informed by the content of the interviews themselves. We searched for common patterns in the coded texts, as well as for factors that could account for the pronounced differences between individuals and between facilities (Gon et al., 2018). To interrogate our themes, we sought counterexamples and alternative explanations in the texts, in the existing literature, and from other projects we have conducted in similar settings. Thus, code and theme development were informed by transcripts, theory, by observations, and by behaviour-change relevance.

The observations were analysed through discussions between team members during and after each day at a clinic. The written notes taken during these observations and discussions were then analysed using the same processes as the interview transcripts described above.

### 2.1 Ethics

The London School of Hygiene and Tropical Medicine Research Ethics Committee and the Zanzibar Medical Research Ethics Committee approved the project. Written consent was sought from interviewees. Permission to visit hospitals was granted by the Ministry of Health Zanzibar. While it was not possible to obtain written consent from every staff member and patient present during the site visits, verbal and written consent was obtained from patients and staff members who were subject to periods of systematic observation.

## 3 Results

### 3.1 Overview of the four facilities

Table 2 summarises the differences between the facilities. Two facilities (A and B) had a higher volume of deliveries and were better equipped while the other two facilities had a lower delivery volume and poorer infrastructure and consumables supply (C and D).

**Table 2:** Overview of facilities, their infrastructure, and consumable availability.

	Facility A	Facility B	Facility C	Facility D
Births per month	350	400	74	95
Piped water	**Yes**	**Yes**	Daily interruptions	None for 7 days
Functional sink in delivery room	**Yes**	**Yes**	No	No
Elbow tap at nearest sink	**Yes**	Poor design^b^	No	No
Disposable drying towels	No	At one sink	No	No
Liquid soap	**Yes**	**Yes**	**Yes**	**Yes**
Hand gel	**Yes**	**Yes**	In storage	No
Delivery sets prepared in advance	No	**Yes**	No	Often incomplete
Clean gloves	No	**Yes**	**Yes**	No
Sterile gloves	**Yes**	**Yes**	**Yes**	Sold in ward
Plastic delivery sheet	From mother	From mother	From mother	Sold in ward
Apron	**Disposable**	**Disposable**	**Reusable**	No
IPC committee	**Yes**	**Yes**	**Yes**	No
Perineum cleaning material	Kanga^a^	Kanga^a^	**Gauze**	Kanga^a^
Orderlies deliver babies	Yes	**No**	Yes	Yes
Sink inside the delivery room	**Yes**	**Yes**	**Yes**	No
Footsteps from handwash sink to delivery beds	**2 to 4**	**5 to 8**	7 to 13	15 to 17 (inc a door)
Footsteps from bed to handwash to gloves to bed	**8 to 13**	**14 to 17**	33 to 34	32 to 34 (inc a door)
Delivery beds	3	3	2	2
Birth attendants per shift	2 to 4	3	2 to 4	0 to 2

*Note.* These data describe the facilities on the week of the visits. Births per month, infrastructural problems, and the availability of consumables will vary over time. Facility characteristics that may facilitate relatively better hygiene or lower infection risk are emphasised in bold typeface.

a Multipurpose rectangular pieces of cotton brought by the mothers; one used as sheeting for the bed during delivery and another used for wrapping the new-born.

b The elbow-opening faucet was small and difficult to use.

### 3.2 Theme 1: how ward layout facilitates or impedes hygiene behaviour

There were substantial differences between facilities in how the layout of the delivery room and the consumables facilitated these hygienic practice. In some facilities, the time costs, energy costs, and mental costs of executing these steps was much higher than in other facilities. For instance, the layout of one facility necessitated a 32–34 step round-trip, including a door, to get from patient to tap to gloves to patient. Few gloves were kept in the delivery room, and an additional 40-step journey to retrieve more from the store cupboard in the next room was often necessary. The consequences of this layout were recognised by staff members as inappropriate, particular in a facility where the sink was outside the delivery room:

“Maybe it is a challenge in our labour room as you have to move here and there, but it could be simple to wash hands if the sinks could be there, so if you put a water sink it will help.” (Nurse birth attendant.)

“It would have been better if the taps were available in every ward, it would have helped very much to make someone not forget to wash hands.” (Midwife).

“If the sinks are available in every room, one cannot leave aside washing hands. However, when the sinks are far, one starts to think of going from here to there so one sees some sort of a burden.” (Nurse birth attendant.)

During fast deliveries, there is insufficient time to find the gloves:

“When your assistant comes to scan the cupboard for [i.e. retrieves] gloves, you have already touched the head of the baby [that has just been delivered].” (Nurse birth attendant.)

**Fig. 2:**
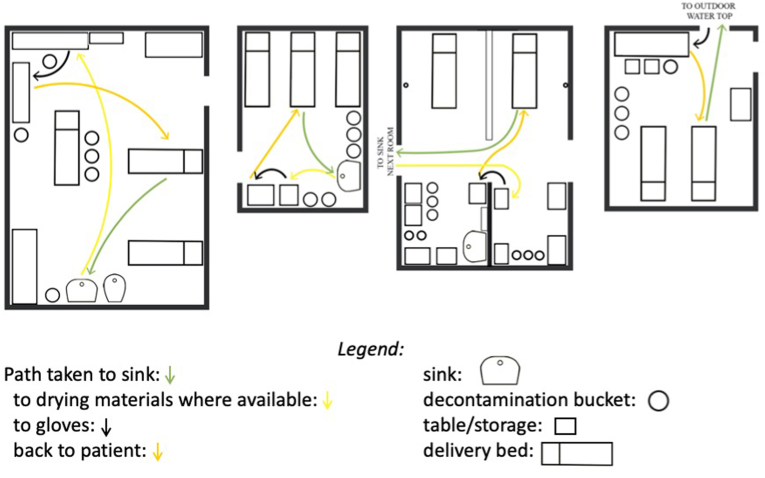
Layout of delivery rooms in the four facilities. To preserve facility anonymity, the plans have not been labeled. Spaghetti plot lines show the pathway taken by a midwife who needs to wash hands and apply gloves while attending to a patient.

The same set of tasks in another facility involved fewer steps (8–13, depending on the bed). However, hand hygiene infrastructure/consumables were not located close to each other, nor were they arranged in the order in which they are typically used. In two facilities, there was no functional sink in the delivery room, and in one, the hand gel was kept in a separate storeroom. Thus, essential hand hygiene resources were invisible and inaccessible. Only in one facility were the sink, soap, drying material (gauze), and gloves kept within five steps of each other. Fig. 2 illustrates this variability in the convenience of the hygiene materials.

The ward manager in facility A, a facility with better ergonomics, was sensitive to how the arrangement of sinks and other consumables can facilitate hygiene:

“There should be enough hand washing stations, soap, and paper towels should be available. There also should be a hand washing station at least after two to three patients’ bed […] unnecessary movements will be reduced. […] [S]inks make it easy to remember to wash hands when observing patients.” (Ward manager.)

Note how the ward manager recognised the role of sinks as physical reminders to engage in hand hygiene. Several birth attendants noted how the poor room organisation impeded performance during demanding periods:

“Maybe just the time, sometimes you are so busy it becomes difficult to go and find water and soap, you might find the mother is fully dilated and the baby is coming out, it becomes difficult to find the soap and wash hands in that situation.” (Nurse birth attendant..)

Theme 2: Attitudes, beliefs, and behaviours relating to consumable use.

Delivery packs: Delivery packs include forceps, blades/scissors for cutting the umbilical cord, a ligature for tying the cord, gauze and cotton swabs. In facility B, these were prepared in advance and wrapped in a sterile cloth, making workload more manageable for midwives just before a delivery, a critical time for hand hygiene. Facility D also prepared delivery packs in advance, but these were often incomplete, requiring birth attendants to search for sterilised tools immediately before or during the delivery. Midwives described discovering mid-delivery that key components were missing. Pre-prepared delivery packs necessitate several full sets of equipment, and the ward manager of facility C listed equipment shortage as a reason delivery packs were not always available. Midwives in Facility B noted the importance of a complete, convenient delivery pack for avoiding hand recontamination after hand hygiene:

“Now if you do not have an assistant, you might take it if you draw that medicine and touch other things, sterility is broken, unless you prepare yourself with all the needed items on a tray close to the delivery bed.” (Nurse birth attendant.)

*Soap and handwashing.* Liquid soap bottles were present at least one sink in all of the labour wards (but sometimes not in the delivery room) during our observations. Birth attendants reported that liquid soap was absent for a few weeks several times a year, forcing the birth attendant to rely on cheaper powder soap that *“dries your skin and causes irritation”* (nurse birth attendant). Bar soap did not appear to be in use.

During observation periods, liquid soap was typically used after “dirty” procedures, where contamination with body fluids had occurred. In interviews, birth attendants often mentioned the importance of handwashing after such procedures for protecting themselves and other mothers:

“There are some women with infections and we as providers can't tell who it is. Therefore, in order not to infect yourself, when you remove gloves you have to wash your hands; and some gloves could be torn without you knowing so it is important to wash hands.” (Nurse birth attendant.)

Observation on the wards suggested that washing of hands *before* aseptic procedures was less frequent than *after* procedures. During interviews, birth attendants described how such handwashing posed no major difficulty for them, except for during emergency deliveries:

“There are emergency situations in which one may forget to wash hands, like when a pregnant woman comes fully dilated in which you just wear gloves and assist her. But that doesn't happen all the time, most women come not fully dilated.” (Nurse birth attendant.)

“Yes, it is important, one can wash your hands, dry them and then wear gloves especially when the situation allows, but when a woman arrives here fully dilated, one just wears gloves.” (Orderly birth attendant.)

However, when asked during the interview to estimate the number out of 10 colleagues that washed hands before a delivery, responses ranged from 0 to 8, with many estimating that about half would wash hands before a delivery. Numeric estimates were similar for vaginal exams. Ward managers similarly understood hand hygiene was less than universal:

“Up to five out of ten nurses can wash hands before a delivery … Eight to ten nurses could wash their hands after a delivery.” (Hospital manager.)

Birth attendants explained this low compliance among peers as a consequence of laziness, lack of education, poor understanding of consequences, forgetfulness, negligence, and, consistent with the quotes above, time constraints.

*Hand gel sanitizer.* Facility B was observed to make its own hand sanitizer and was the only facility where it was readily accessible and often used. While there were no religious concerns among the largely Muslim staff about alcohol gel use, there appeared to be some doubt about its effectiveness. One midwife described how it might be appropriate to use “*when we want to do minor procedures, but not during delivery*”. Another birth attendant described it as *useful in an emergency* rather than as a standard replacement for water/soap before aseptic procedures:

“We use soap and water if we see that there is still time before a mother delivers, if the mother is almost ready to deliver when she comes then we use hand gel.” (Nurse birth attendant.)

*Drying materials and hand drying.* Wet hands are difficult to glove, and the sensation of wearing gloves over wet hands is unpleasant. During the observation periods, we noted that the absence of convenient, disposable hand drying materials created difficulty for the birth attendants. We observed air drying of hands (which can take several minutes) and as well as the use of personal handkerchiefs, cotton gauze, or the front of the uniform to dry hands. Birth attendants mentioned that staff members do not wash hands before a vaginal exam *“since they don't have drying materials*” (Midwife.)

*Gloves and their use.* During our observations, glove use during aseptic procedures was universal, but contamination of gloved hands was common.

Birth attendants sometimes layered multiple pairs of gloves, removing the top layer after one procedure and continuing to the next procedure using the inner layer. This layering of glove use was observed in multiple facilities and described by multiple birth attendants. There were differences in when the top layer is removed with some shedding the top layer to “receive the child” and most others shedding to cut the umbilical cord. Birth attendants also reported layering gloves so that they could efficiently attend to multiple patients.

Contamination of gloved hands through contact with potentially infective surfaces was common during observations. In interviews, birth attendants mentioned that contact with objects such as tables, drawer handles, the mother's kanga, the injectable oxytocin, the drip, unsterilised Cheatle forceps, and syringe boxes, as well as the mother was common and that they could lead to infection transmission. While some midwifes made relatively little effort to avoid recontamination, others tried but were often unsuccessful. Our observations illustrate this:

While the woman is getting down from the bed, the mackintosh falls down on the floor.

Nurse A picks the mackintosh up with her sterile gloves on (while doing so, she is observed struggling not to touch the floor but she touches it a little bit).

Or:

The birth attendant puts on two pairs of sterile gloves and asks the mother to lay in a proper position. She uses the sterile gloves wrapping to hold the mackintosh and put it properly.

At facility B, nurse birth attendants reported on how preparation can prevent glove recontamination:

[to avoid contamination] you have to prepare yourself well; when a mother is about to deliver before wearing gloves you put all equipment in place. We have folded the delivery sets on green towel so that each worker can use a set which is complete and not the set with missing equipment, this will avoid one from looking for thing unnecessarily.”

*Delivery surfaces: kanga and mackintosh.* Delivery beds were covered with a mackintosh (a plastic sheet), which was covered with a kanga (a multipurpose cotton rectangle) during labour and delivery. These were both brought to the facility by the mother. Kangas were brought from home while mackintoshes were purchased from nearby pharmacies. Selling mackintoshes to mothers was discouraged by some managers who were concerned about accusations that facility gained from sales: “*trouble comes in when she sells the things to a person who feels that the equipment is available, but it is being sold to her”* (Ward manager, facility A).

After the delivery, the woman's kanga was used to clean the vagina/perineum in three of the four facilities (facility C used cotton gauzes). The use of often-soiled kanga to clean the vagina after birth may pose a significant infection risk. After the placenta had been delivered, another kanga was sometimes used as a makeshift sanitary pad. A separate kanga was also used to wipe clean and then wrap the baby after delivery.

### 3.3 Theme 3: social and managerial influences on hygiene

*Social norms and social sanctions.* Birth attendants reported that hand hygiene compliance among colleagues was often low. Birth attendants also reported that negative consequences for those who do not handwash were generally absent:

Interviewer: “*Have you heard of any complaints about health providers who do not wash hands before assisting women to deliver?”*

Orderly birth attendant: *“I have never heard of such complaints, not only from here but from other hospitals as well, no woman has complained of being attended by a doctor who didn't observe hand hygiene while assisting mothers during delivery”.*

Sanctioning was seen by birth attendants as demeaning and childlike with one midwife in facility D reporting that *“We do not give punishments because we are all adults, we just remind each other.”* One midwife in facility D hinted at how loyalty to one another precluded reporting poor hygiene:

Interviewer: *“Have you ever reported your colleague that he/she is not washing hands?”*

Midwife: *“There are no such customs and there is a habit of looking after one another.”*

Indeed, in all facilities, we observed a notable degree of mutual respect between staff members of different cadres. Senior staff members treated all staff, including orderlies, with politeness and kindness.

*Facility organisation and management.* Several managerial/organisational characteristics appeared to distinguish poorer performing facilities from better facilities. In facility B, staff members were given specific tasks by their superiors (e.g., prepare six delivery packs) in the morning. In the other facilities, the division of roles was less clear. The specificity of roles and the fact that named individuals took responsibility for their completion may have contributed to the better organisation observed in facility B.

Another distinguishing feature of facility B was the “hands-on” approach of the hospital manager. She was observed, for example, mopping the floor and engaging in other cleaning activities. In the interview, the manager described how she led by example. She also visited the maternity ward daily and relayed detailed observations on the quality of care to us. While it is difficult to gauge if the observed behaviour is representative, the midwives in that facility also noted that the facility management prioritised hygiene. This stands in contrast to other hospital managers who appeared to make more perfunctory visits to the maternity ward. Some managers explicitly regarded hand hygiene as an issue for staff members. Asked if there are reminders for handwashing, a ward manager responded: “*We do nothing; it is a person's concern.”* (A summary of the major findings is presented in Table 3.)

**Table 3:** Summary of modifiable factors contributing to lower hygiene rate and higher infection rate.

Contributing factor	Processes by which factor influences infection rate	Potential solutions
Layout of delivery ward	The delivery room impedes or encourages hand hygiene by making sinks etc. accessible and noticeable or inaccessible and out of sight, respectively.	1. One-off infrastructural changes improve hygiene-related ergonomics.
Lack of time for hygiene during high-intensity periods.	During periods of high intensity, hand hygiene is forgone due to competing priorities.	1. Prepacked delivery packs to alleviate workload at critical moments. 2. Hand gel for efficient hand hygiene before procedures. 3. Drying materials so ensure hands can be quickly dried.
Recontamination of hands after hand hygiene and glove application.	Recontamination of hands following hand hygiene was common and may increase the infection rate.	1. Prepacked delivery packs would reduce the need for contact with objects as part of delivery preparation. 2. Reduce glove scarcity so that birth attendants are not incentivised to retain contaminated gloves.
Use of Kanga as a delivery surface and a to clean perineal area following delivery.	Kangas may not be adequately clean before the delivery and often are contaminated during the delivery. Their use to clean the mother after delivery constitutes an important infection risk.	1. Provide and use cotton gauzes for post-delivery cleaning.
Acceptance of variance in hygiene standards among birth attendants.	Individuals with who engage in less hygiene do not experience many social sanctions or influence from other staff members.	Staff who invest more in hygiene may be emboldened to influence others if: 1. the negative consequences of hygiene for mothers and the broader community are stressed. 2. management demonstrates commitment to hygiene by, e.g., investing in consumables and infrastructure or regular audits.

## 4 Discussion

This study sought to investigate how variability in the ward layout, organisation, staff beliefs, relate to hand hygiene through a series of interviews and observations in four facilities. Two of the facilities had both a higher volume of deliveries and a higher rate of hand hygiene compliance (facilities A and B) compared to the other two (C and D). In the following sections, we discuss what factors may account for the differences between individuals and facilities and what this means for infection prevention strategies in Zanzibar and beyond.

### 4.1 Delivery room organisation and layout make hygiene cognitively taxing and time-consuming

When hand hygiene is time-consuming, it is likely that birth attendants will engage in it less frequently. Birth attendants have many demands on their time and attention and whether they choose to spend time on hand hygiene, or some other important task should depend on the time/energy costs of a given handwash. Findings from Deyneko et al.’s (2016) cross-sectional study of hand hygiene in Canadian hospitals resonate with this argument. They found that the likelihood of hand hygiene decreased by 10% with every additional meter between the staff member and the sink. The hygiene facilities examined in these facilities in Zanzibar make hand-hygiene expensive in terms of time and energy. Appropriate hand hygiene involved long round trips around the delivery room (ranging from 8 to 34 steps) and – in two facilities – trips to different rooms. The absence of drying materials adds time cost: staff must air dry hands, which may take 2 min or more - or find an alternative drying material. Hand towels, on the other hand, dry hands in about 10 s. We roughly estimate that the absence of drying material and a convenient sink and pair of gloves can add between 30 s to 2 min to every hand hygiene event.

A similar absence of towels for hand drying was noted in 9 of 10 maternity wards studied in Cambodia (Bazzano et al., 2015) and all six wards in a study of post-natal care in Nigeria (Nalule et al., 2020). The more general issue of hygiene ergonomics has not received much attention, however. Few studies in Esteves Mills et al.‘s review of descriptive research on determinants of hand hygiene in low- and middle-income countries examine the issue (Esteves Mills et al., 2020), with for example Chinese midwives noting time constraints, perhaps due to layout (Ji et al., 2005). More studies (22) examined the presence or absence of hygiene materials than the convenience and ease of use of these materials.

There is a *cognitive* as well as a time cost imposed by the layout of the delivery rooms. Seeing an object in the right place at the right time can remind one of the appropriate next step in a sequence of actions (Kirsh, 1995). In the case of hygiene, seeing the gloves when one reaches the hand towels will remind one to don gloves now. Appropriate structuring of the environment can ease the cognitive costs by offloading planning tasks (*what do I do next?*) and search tasks (*where are the towels?*) onto the environment. The cognitive costs of planning/searching in hand hygiene tasks are not trivial since hygiene tasks occur many dozen times per day and are especially critical during emergencies when cognitive resources are allocated to solving other complex problems (Kirsh, 2000). As ecological psychologists have highlighted, careful structuring of the environment such that objects physically and mentally convenient facilitates tasks like hand-hygiene (Hutchins, 2010; Kirsh, 1995, 2000).

### 4.2 Improving delivery room layout and consumable access

One promising way to increase hand hygiene rates is to rearrange consumables so that these practices take less time and energy as well as less planning and searching. For example, placing soap, disposable hand-towels, and gloves close to one another and close to the sink may be an efficient way reduce the temporal and cognitive costs of hand hygiene and to exploit the capacity of objects to cue actions.

Sustained behaviour change is more likely if birth attendants contribute to changes to the ward layout. Evidence from an extensive systematic review by Rowe et al. (2018) suggests that such “group problem solving” is a promising approach for improving health care provider practices and getting health workers buy-in in lower- and middle-income countries. Midwives’ experience of working in the environment means they are uniquely positioned to identify changes that make hygiene more convenient without making other essential tasks more inconvenient.

While the ergonomics of hand-hygiene in hospitals has been examined (Hammond et al., 2014; Suresh and Cahill, 2007), we know of no randomised controlled trials testing the effects of layout or consumable changes on hand hygiene rates or infection rates in delivery rooms. None of the 31 intervention studies documented a recent review of clean birth determinants (Esteves Mills et al., 2020) focus on ward ergonomics, though the WHO Safe Birth Checklist intervention includes a “check” for gloves and water and soap/rub “at the bedside” (WHO, 2015). However, an observational study in Canadian facilities found a strong negative association between sink proximity and handwash probability (Deyneko et al., 2016) while a UK study found greater handwashing rates when sinks were visible (Cloutman-Green et al., 2014). A qualitative study of healthcare facilitates in Vietnam (Salmon and McLaws, 2015) found that reduced access to functional sinks and relevant materials, including hand towels was a barrier to handwashing. Evidence that providing hand sanitizer to health care workers increases hand hygiene is mixed with some studies (Munoz-Price et al., 2014) but not others (Haas and Larson, 2008) showing positive effects. However, given the high time/effort costs of handwashing with soap and water in the settings studied here, hand sanitizer is likely to be a well-used consumable in Tanzanian maternity wards.

The importance of understanding how the environment in which behaviour unfolds is also emphasised by *behaviour setting theory* (Aunger and Curtis, 2016; Curtis et al., 2019). Aunger, Curtis, and colleagues argue that what they term *props* (consumables like soap or drying materials) and infrastructure (sinks or tables) buttress particular behaviour patterns. Interventions that change these props or infrastructure can lead to sustained behaviour change because behaviour is often automatic and habitual response to these elements of the environment. However, such interventions need to be rooted in a detailed understanding of the interaction between behaviour and environment (Curtis et al., 2019).

### 4.3 Knowledge and skills

Participants’ general knowledge of when and how to perform hand hygiene was good. A quantitative study from the same project found that while knowledge of hand hygiene did predict somewhat higher compliance rates, hand hygiene was low in groups with and without this knowledge (Gon et al., 2020). Therefore, interventions targeting birth attendant knowledge alone may not be a promising path. This conclusion is consistent with other studies showing that neither educational interventions without substantial learner-engagement (Rowe et al., 2018) nor printed educational materials (Rowe et al., 2005) have strong positive effects on health care worker behaviour. Other studies show good knowledge but poor compliance in other settings (Nalule et al., 2020). One exception may be knowledge related to beliefs in the effectiveness of handgel: The interviews suggest that midwives may be somewhat sceptical about its value and if these views are widely held, interventions targeting relevant gel-related knowledge and attitudes may also be beneficial.

### 4.4 Changing social norms

Birth attendants themselves recognise that colleagues often do not perform hand hygiene before aseptic procedures, and this creates additional challenges for hand hygiene interventions. The social science literature (Bicchieri and Xiao, 2009; Nolan et al., 2008) suggests that such descriptive norms (i.e., one's beliefs about the other's actions) have a strong influence on behaviour. This norm psychology will tend to exacerbate problems in poorly performing facilities since birth attendants will follow typical patterns of non-compliance. There are few if any studies on the role norms as determinants of hygiene in maternity wards (Esteves Mills et al., 2020). It may be fruitful to examine if providing information about hand-hygiene rates in other better-performing facilities or wards can ameliorate these effects of these descriptive norms. Interventions that enable management and midwives to demonstrate a commitment to improving hygiene may also help shift norms.

### 4.5 Limitations and strengths of current study

One limitation of this study was that we focused on hand hygiene and excluded other infection-relevant behaviours like clinical waste disposal and equipment sterilisation as well as a range of other delivery practices important for mothers' and newborns’ health. It is, of course, important to avoid changes to delivery rooms that improve hand hygiene at the expense of other important objectives, including the emotional wellbeing of the patients and staff. While few of the changes suggested here are likely to have adverse effects in these areas, we recognise that improved design of labour wards may need to accommodate a broader set of priorities than just infection control (Foureur et al., 2010; Tunçalp et al., 2015).

A second limitation of the study is that normative behaviour like hand-hygiene is typically subject to social desirability biases. In other words, birth attendant's behaviour, as well as their reflections during interviews, are likely to be shaped by their desire to create a good impression or to satisfy what they believe to be our expectations. While we tried to limit these biases by, for example, describing the goals of the project in broad terms, readers should interpret our results with this limitation in mind.

In retrospect, it may have been useful to make more detailed physical measurements of the wards and delivery rooms (e.g., area of rooms, distances in meters rather than steps). While we doubt the results would have been qualitatively different had we recorded this data, these kinds of precise measurements would have enabled comparisons with other studies and settings.

With just four facilities and a subset of people within each one, we cannot draw any firm conclusions about the causes of different hygiene rates. The problem is exacerbated by the fact that many of the “good” things were common in the better-performing facilities and missing from the poorly performing facilities (IPC committees, better management, better layout, better consumable supply, more engaged management, ergonomics). It is unclear how well these findings and recommendations generalise to other facilities in Tanzania or indeed lower-incomes settings across the globe.

A strength of the study is that it provides a rare and detailed exploration of how hand hygiene and delivery ward organisation/layout interact in a low-income setting. As Ulrich et al. (2008) note, “the neglect of human factors and research methods are major weaknesses of handwashing research and the infection control literature in general”. The study is also unusual in its broad and detailed approach which included interviews with birth attendants, management, cleaners, as well as observations of multiple deliveries and other procedures by researchers with both medical and social science training. Finally, a strength of the study is that it has brought attention to several plausible intervention targets. We conclude by summarising these.

## 5 Conclusions

Our results suggest several potential ways to make hand hygiene easier to perform and less time-consuming through relatively low-cost changes to maternity wards. Providing personal supplies of antiseptic hand gel and locating hand washing facilities, including disposable hand towels, in places that fit with workflow at the time of delivery are promising interventions. Such changes could substantially reduce the time and effort needed to maintain compliance with hand hygiene standards without imposing undue time costs on staff members and deterioration of patient care.

## Credit author statement

Mícheál de Barra: Writing - Original Draft, Formal analysis, Investigation. Giorgia Gon: Conceptualization; Formal analysis, Writing - Review & Editing, Investigation. Susannah Woodd: Conceptualization, Formal analysis, Writing - Review & Editing. Wendy J Graham: Conceptualization, Supervision, Writing - Review & Editing. Marijn de Bruin: Conceptualization, Supervision, Writing - Review & Editing. Catherine Kahabuka: Investigation, Formal analysis. Jess Williams: Formal analysis. Khadidja Konate: Visualization. Said M Ali: Project administration, Investigation, Conceptualization. Rukaiya Said: Project administration. Loveday Penn-Kekana: Investigation, Supervision, Writing - Review & Editing.
